# Alexithymia, emotion regulation and autistic traits in Familial adenomatous polyposis

**DOI:** 10.1192/j.eurpsy.2024.755

**Published:** 2024-08-27

**Authors:** A. Vieira, F. Martins, A. S. Machado

**Affiliations:** Psychiatry, Centro Hospitalar Universitário de São João, Porto, Portugal

## Abstract

**Introduction:**

Familial adenomatous polyposis (FAP) is a condition characterised by multiple polyps inside the colon or rectum, leading to colorectal cancer in all patients who do not perform prophylactic colectomy and a higher risk of cancer in other organs. Nevertheless, it has been reported that 14-48% of patients do not comply with regular endoscopic surveillance, which seems to be related to the lower levels of emotional distress observed in these patients. Also, APC gene polymorphisms have been described as being related to neurodevelopmental disorders, such as autism.

**Objectives:**

To study the prevalence of alexithymia, autistic traits and emotion regulation strategies in patients with FAP.

**Methods:**

We conducted a cross-sectional study of patients with a genetic or clinical FAP diagnosis and assessed for alexithymia, autistic traits and emotion regulation using psychometric tests - Toronto Alexithymia Scale - 20 items (TAS-20), Autism-Spectrum Quotient Test (AQ) and Emotion Regulation Questionnaire (ERQ), respectively. The control group were patients with Lynch Syndrome. Statistical analysis was performed using SPSS vs.26.

**Results:**

We recruited a total of 20 patients (10 with FAP vs 10 with Lynch Syndrome). Nine patients were male (45%) versus 11 female (55%). The mean age was 53,35 years (SD 18,4). Half the sample presented a low educational level (equal or inferior to 4th grade).

The overall prevalence of alexithymia was 65%, with an 80% prevalence in FAP patients and 50% in Lynch Syndrome. TAS-20 total score was higher in FAP patients (69,0 vs 60,7; p=0,68). Externally-oriented thinking subscale score was statistically higher in FAP patients (p=0,024).

The overall prevalence of autistic traits was 25%, and the mean AQ score was higher in FAP (23,4; SD 4.97) compared to Lynch Syndrome patients (20,2; SD 5.57), but there were no statistically significant differences between the diagnoses (p=0,192).

A moderate positive correlation exists between Total AQ and Total TAS (r=0.51; p=0.020).

Concerning the scores obtained on the ERQ scale, most participants (14; 70%) use Expressive Suppression as a regulation strategy. Patients with Lynch Syndrome had higher scores than those with FAP, both in the Cognitive Reappraisal (4.22; SD 1.58 vs 4.28; SD 0.90) and Expressive Suppression (4.58; SD 1.08 vs 5.15; SD 1.03) domains.

The average AQ score for patients who mostly use expressive suppression is significantly higher than for those who use cognitive reappraisal (23.86 (3.63) vs 17.00 (6.6); p=0.039).

**Conclusions:**

The preliminary results of this study point to high levels of alexithymia and autistic traits in this population, and a higher tendency to regulate emotions by expressive suppression.

The main limitation of the study was the small sample size, which reduced the power of the study to find statistically significant differences. Also, in future studies, a different control group should be considered.

**Disclosure of Interest:**

None Declared

